# Development, delivery, and evaluation of the Texas Epidemic Public Health Institute infection control module program 200 series

**DOI:** 10.3389/fpubh.2025.1599312

**Published:** 2025-07-03

**Authors:** Kayla E. Ruch, Anabel Rodríguez, Janelle Rios

**Affiliations:** ^1^Texas Epidemic Public Health Institute Infection Prevention and Control Program Manager, School of Public Health, The University of Texas Health Science Center at Houston, Houston, TX, United States; ^2^Department of Epidemiology, The University of Texas Health Science Center at Houston, Houston, TX, United States; ^3^School of Public Health, Texas A&M University, College Station, TX, United States; ^4^Department of Environmental and Occupational Health, Texas A&M University, College Station, TX, United States; ^5^Environmental and Occupational Health Sciences, Texas Epidemic Public Health Institute, The University of Texas Health Science Center at Houston, Houston, TX, United States

**Keywords:** infection preventionist, health and safety training, occupational health, healthcare-acquired infections, IPC

## Abstract

**Background:**

The Texas Epidemic Public Health Institute (TEPHI) aims to safeguard public health and the Texas economy by preparing for infectious disease outbreaks. The Infection Prevention and Control (IPC) Webinar series was created to offer free educational resources and continuing education for public health and healthcare personnel responsible for IPC programs in rural regions of Texas. The IPC 200 Series succeeds the founding IPC 100 Series established by the TEPHI Small Rural Healthcare Preparedness.

**Methods:**

IPC registration and attendance data were collected through WebEx® and Microsoft Teams®, which also served as the platforms for module delivery. Learning assessments and post-module evaluation surveys were administered using QuestionPro®. Module content was developed using resources adapted from the Association for Professionals in Infection Control and Epidemiology (APIC), the Occupational Safety and Health Administration (OSHA), the Centers for Disease Control and Prevention (CDC), The Joint Commission (TJC), and the Centers for Medicare and Medicaid Services (CMS). The Kirkpatrick Model assessed knowledge effectiveness through knowledge activities, post-evaluations, and a completion impact survey.

**Results:**

IPC 200 Series had 1,088 attendees to live modules and generated >4,400 YouTube views. Each module was accredited for 1.0 hour of public health education and IPC certification (a-IPC), with eight of ten sessions offering 1.0 continuing education hours for certification in infection control (CIC) for infection preventionists. Of 286 participants completing post-knowledge assessments, the average score was 91.0% (Range: 81.0–96.0%). Post-evaluations (*n* = 271) rated the content highly (mean: 4.8/5.0) for beneficial, easy to understand, and clear/concise. Additionally, 90.4% of respondents indicated plans to implement the knowledge gained, and 98.9% expressed interest in attending future sessions.

**Conclusion:**

IPC series improved participants’ knowledge of infection prevention and control best practices. By disseminating evidence-based education and providing no-cost continuing education, the series equipped healthcare personnel with the tools to foster safer environments for patients and staff in healthcare settings.

## Introduction

The field of infection prevention and control (IPC) utilizes evidence-based approaches to prevent patients and healthcare workers from being harmed by avoidable infections (1). Effective, efficient, and operational IPC measures require constant actions from the entire healthcare organization and system, including policy development and support from organization managers, healthcare workers, and patients. Infection prevention and control is a unique field of practice for patient and healthcare workers’ safety (1, 2). Globally, seven patients in high-income countries and 15 in low- and middle-income countries out of 100 hospitalized patients develop healthcare-associated infections (HAIs) during acute-care hospitalization (2). The 2024 World Health Organization (WHO) Global Report on Infection and Control, as well as the Organization for Economic Co-operation and Development (OECD), estimated that nearly 3.5 million people are at risk of death due to HAIs between 2015 and 2050, with 136 million infections resistant to antibiotics annually (2, 3).

According to the WHO 2024 Infection Prevention and Control Report, the results of a detailed global survey on the minimum requirements for national IPC programs conducted by WHO in 2023–2024 revealed that 71.3% (107 of 150) of countries had an active national IPC program, defined as a functioning program with an annual work plan and budget (2). The survey highlighted areas of advanced implementation and gaps requiring further improvement in national IPC programs. Significant discrepancies were observed across income levels, with high-income countries reporting more robust implementation. However, critical gaps remain in budget allocation, training, healthcare-associated infection surveillance, and monitoring systems, particularly in low-income countries (2).

Infection preventionists (IPs) are healthcare professionals dedicated to implementing and overseeing infection prevention and control strategies within healthcare settings to ensure the safety of patients and healthcare workers through cost-effective policies, procedures, and resource utilization (4). These professionals, originating from diverse disciplines such as nursing, epidemiology, public health, microbiology, and medicine, play a critical role in reducing HAIs (4). In the United States (US), approximately one in every thirty-one hospitalized patients develop at least one HAI during admission (5). IPs core responsibilities include surveillance of infection patterns, evaluation of infection control practices, education of healthcare personnel, provision of evidence-based guidance to institutional leadership, analysis and reporting of infection data, development of policies and protocols, and coordination with public health authorities at local, state, and national levels (4). Additionally, IPs are entrusted with developing, implementing, and continuously analyzing infection prevention and control programs within healthcare settings (4).

Texas spans across 254 counties and has over 31 million residents (6). Census projections predict that the population could surpass 45 million by 2040 (6). This anticipated growth is expected to have significant implications for infrastructure development, healthcare systems, educational services, and political representation (6). The Texas Epidemic Public Health Institute (TEPHI), a state agency of higher education headquartered at The University of Texas Health Science Center at Houston (UTHealth Houston) School of Public Health, was established to address critical public health needs (7). TEPHI’s mission is to support a strong economy by enhancing the capacity and resilience of Texas communities to respond effectively to future infectious disease outbreaks (7). TEPHI focuses on strengthening and supporting a well-trained public health workforce, preparing communities for public health threats related to infectious diseases, and fostering a resilient state economy capable of withstanding such challenges (7). Texas Senate Bill (S. B.) 1780 was passed in May 2021 with bipartisan support by the 87th Texas Legislature, establishing TEPHI to prepare Texans to mitigate the impact of infectious diseases due to the COVID-19 pandemic and any future epidemics (7). The organization is built on four program pillars: early detection, public health communication, a public health reserve network, and training initiatives (7).

The IPC program—and its webinar module series—sits within TEPHI’s integrated public health reserve network. This webinar module series was developed to provide small rural hospitals with infection prevention education and readiness training and equip marginalized communities with accurate, scientifically sound resources to prevent and help mitigate the impact of infectious diseases, thereby avoiding overwhelming small rural hospital capacities (7). This program was developed to provide no-cost infection prevention and control education to IP-designated personnel, with a special emphasis on rural and low-resource healthcare facilities. The purpose of this paper is to (1) review the second year of the Infection Control series by analyzing module registration information, attendance numbers, YouTube views, and post-survey evaluations, (2) identify strengths and opportunities for improvement, and (3) provide recommendations for year three of the TEPHI Infection Prevention and Control module series.

## Materials and methods

The TEPHI Infection Prevention and Control module series was based on the eight core components of the Certification in Infection Control and Epidemiology (CIC®), developed by the Certification Board of Infection Control and Epidemiology (CBIC), Inc. (8, 9). The CIC® examination serves as an industry-standard metric, assessing the core knowledge, skills, and abilities essential for infection preventionists in the US and other countries (8, 9). While obtaining certification is optional, it is regarded as the benchmark for best practices in the field in the US. The eight core components include: (1) identification of infectious disease processes, (2) surveillance and epidemiology investigations, (3) preventing and controlling the transmission of infectious agents, (4) employee/occupational health, (5) management and communication, (6) education and research, (7) environment of care, (8) including cleaning, sterilization, and disinfection, and asepsis (8, 9).

The series utilized the Kirkpatrick Model with Level 1 reaction captured by asking participants to complete a post-module satisfaction evaluation, Level 2 learning with each model having a learned activity, and Level 3 behavior inviting individuals that attended to complete a series impact survey in December 2024 (10). Specific topics and subtopics were selected based on participant responses to post-evaluation surveys from the TEPHI Infection Prevention and Control 100 series modules delivered in the program’s first year or pilot year (11). IPC registration and attendance data were collected through WebEx® and Microsoft Teams®, which also served as the platforms for module delivery (12–15). Learning assessments and post-module evaluation surveys were administered using QuestionPro® (12–15). Participant demographics were gathered during registration and included names, email addresses, credentials, organizational affiliations, job titles, and years of experience in infection prevention and control in WebEx® and Microsoft Teams® (12, 14). Modules 201–207 were delivered via WebEx®, and Modules 208–210 were delivered on Microsoft Teams® (12, 14). Registration, attendance, and YouTube viewer data were analyzed using Stata/SE version 17 (16). The data was presented as descriptive statistics. A complete case analysis was performed on registration data, producing a sample size of 2,643 to 2,596 to measure central tendency metrics using Stata software (16) *t*-statistics and *p*-values were calculated from the 100 and 200 series comparison. A *p*-value of <0.05 was used to measure level of significance.

The module series incorporated resources, recommendations, and regulations from the Association for Professionals in Infection Control and Epidemiology (APIC), the Occupational Safety and Health Administration (OSHA), the Centers for Disease Control and Prevention (CDC), The Joint Commission (TJC), and the Centers for Medicare and Medicaid Services. Additional evidence-based practices and resources were selected from the CIC exam, and the material was requested from participants from the previous year’s pilot module series. Ten 1-hour modules were developed to cover multiple components. Within the one-hour module, educational material was presented for 35–45 min, learning activities for 10–15 min, and concluded with a question/answer session for participants. To pass the module, participants must obtain 80% or higher on the learning activity. Learning activities were developed in and completed Qualtrics® and QuestionPro® (13, 15). Multiple platforms were used due to changing university licensure agreements. Questions and answers on each platform were coded the same.

All modules were provided at no cost, recorded, and uploaded onto the TEPHI YouTube channel. Presentation slides and module links were shared with attendees for future reference. Each module’s informational flyer was created and promoted through the TEPHI communications network and distributed to individuals on social media platforms such as Instagram, X, and LinkedIn. Module material focused on Texas-specific regulations and recommendations; however, registration was not restricted to Texan residents. Individuals around the country and globally could register and view the material. Table 1 provides an overview of the modules. Modules 203–210 were approved for 1.0 continuing education credit hours from the National Board of Public Health Examiners (NBPHE) for individuals with public health certification (CPH®) and the Certification Board of Infection Control (CBIC) for individuals with CIC® or a-IPC® certifications (17, 18).

**Table 1:** Infection control series module overview.

Module Number and Title	Overview	Learning Objectives	Additional Material
Module 201: Epidemiology	A brief introduction of the Epidemiology terminology, models, and concepts	Explain the study of epidemiology. Explain epidemiological models in basic terms. Define the terms “Sensitivity” and “Specificity.”	Epidemiology textbooks, Epidemiology recommendations from CDC and APIC (26)
Module 202: Occupational Epidemiology and Prevention	A brief introduction of occupational epidemiology and OSHA Bloodborne Pathogen Exposure Control Plan.	Explain how occupational epidemiology concepts are used in infection prevention. Apply the OSHA Bloodborne Pathogen Exposure Plan.	OSHA Bloodborne Pathogen Plan and Regulations, APIC textbook (27)
Module 203: Surveillance	A brief introduction on what surveillance is, when, and how to conduct surveillance activities.	Define the term, “surveillance.” Explain the purpose of conducting surveillance, including timing. Develop and apply a surveillance plan.	APIC textbook and Texas Department of State Health Services (DSHS) resources (28)
Module 204: Data Handling	A brief introduction to infection prevention and control data practices	Identify the types of data to collect. Explain the technique known as data cleaning. Summarize and present collected data.	APIC Textbook, Epidemiology and statistical methods resources (29)
Module 205: Hemodialysis	A brief introduction to hemodialysis, monitoring practices, and CLABSI risks	Define hemodialysis. Explain monitoring and auditing practices. Outline reasons why hemodialysis is a CLABSI risk.	APIC Textbook, National Healthcare Safety Network Manual, and Association for the Advancement of Medical Instrumentation resources (30)
Module 206: Emerging Infectious Diseases	A brief introduction to Mpox, *C. auris*, and H5N1	Apply IPC practices to the following pathogens: Mpox, *C. auris*, and d H5N1.	APIC textbook and recommendations, CDC guidelines/recommendations, WHO recommendations, Texas DSHS guidelines/recommendations (31)
Module 207: Outbreak	This is a brief overview of how to identify an outbreak situation and conduct an outbreak investigation in a healthcare setting by implementing infection prevention strategies, ensuring patient and staff safety, and maintaining public health standards.	Identify the elements of an outbreak in a healthcare setting. Perform a simple mock outbreak investigation. Outline a case example.	APIC textbook, CDC guidelines, and Texas DSHS guidelines/recommendations (32)
Module 208: Contact Trace	An overview of contact tracing during an outbreak or exposure event in a healthcare facility, with a focus on strategies to reduce transmission and ensure the safety of patients and staff.	Explain the technique known as contact tracing. Identify conditions in which contact tracing should be employed. Outline a case example.	APIC Textbook, CDC and WHO contact tracing recommendations (33)
Module 209: Infection Prevention and Control Collaboration	A brief introduction of collaborative processes involved in infection prevention and control (IPC) across all areas of a healthcare facility.	List common IPC department collaborators. Demonstrate techniques to obtain stakeholder buy-in and support. Demonstrate approaches to obtain IPC support with staff and patients.	APIC Textbook, CDC communication recommendations, WHO communication recommendations, and Joint Commission regulations (34)
Module 210: Quality Improvement	This introduction will cover selecting quality improvement projects, collaborating across departments, and establishing KPIs to enhance infection prevention and control.	Identify opportunities for quality improvement activities. Select a quality improvement project based on available opportunities. Demonstrate methods to collaborate with multiple departments on quality projects. Propose key improvement indicators (KPIs).	APIC Textbook, CDC recommendations, Joint Commission Recommendations, National Institute of Medicine resources (35)

Each participant was asked to complete anonymous post-evaluation surveys after each module in Qualtrics® or QuestionPro® (13, 15) via QR codes that were shared at the end of each webinar and follow-up emails with links to the survey. The survey included 14 questions: 12 closed-ended questions using Likert scales that rated modules on content clarity, usefulness, and impact, and two open-ended questions seeking feedback for future topics and additional feedback or comments. After accounting for missing responses, the initial sample size of 285 participants was reduced to 271 participants. Descriptive analyses of central tendency metrics and *t*-statistics with *p*-values were conducted using Stata/version SE 17 for a secondary comparison analysis between years 1 and 2 of the TEPHI IPC program (16). Year 1 (100 series) began in March 2023 and ended in November 2023; Year 2 (200 series) started in February 2024 and ended in November 2024. Survey data informed the development of year three infection prevention and control content; year three started on February 2025.

Furthermore, in December 2024, all participants who participated in either year 1 (100 series) or 2 (200 series) were invited to complete an additional survey anonymously via QuestionPro (15). This survey gathered demographic information, feedback on the module series, whether materials were revisited, and current infection prevention and control challenges. Responses also contributed additional insights aimed at enhancing the module series. In conjunction with feedback from the module post-evaluation, this data informed the development of the third year (300 series) of the IPC webinar series.

The 87th Legislature, 2021 Reg, funded this pilot project. Session. The authors reported no potential conflict of interest. Institutional Review Board (IRB) approval was not required since the information gathered was for program evaluation and enhancement; the education series was created using publicly available materials and did not include research with human subjects or protected health information.

## Results

The TEPHI IPC Webinar 200 Series registered 2,635 participants, drew 1,088 attendees, and accumulated 4,487 YouTube views as of May 2025. Of those registrants, 2,146 (81.4%) had provided a Texas zip code, 489 (18.6%) provided a non-Texas zip code or indicated another country such as Canada, the Czech Republic, the United Arab Emirates (UAE), Egypt, Ghana, India, Nigeria, Pakistan, and Qatar. Module 207: *Outbreak* recorded the highest number of registrations (592), attendees (187), and YouTube views (1,087), as shown in Table 2. In contrast, Module 208: *Contact Trace* exhibited the lowest overall engagement, with 174 registrations, 147 attendees, and 86 YouTube views. Attendance was generally higher in the latter half of the series (Modules 206–210) compared to the first half (Modules 201–205). However, YouTube viewership was consistently higher in the initial half of the series, except Module 208, where views were approximately 2.5 to 3.5 times lower than any other module.

**Table 2:** Modular registration, attendance, and YouTube views.

Module	Date Presented	Registrants	Attendees	YouTube Views (05/31/25)	Total View (Attendee+ YouTube)	Continued Education Eligibility
Module 201	08-Feb-2024	66	46	418	478	CPH, a-IPC
Module 202	07-Mar-2024	129	51	489	578	CPH, a-IPC, CIC
Module 203	04-Apr-2024	235	104	437	559	CPH, a-IPC, CIC
Module 204	02-May-2024	311	100	481	605	CPH, a-IPC
Module 205	13-Jun-2024	286	85	545	649	CPH, a-IPC, CIC
Module 206	11-Jul-2024	439	112	474	610	CPH, a-IPC, CIC
Module 207	01-Aug-2024	592	187	1,087	1,288	CPH, a-IPC, CIC
Module 208	05-Sept-2024	174	147	86	242	CPH, a-IPC, CIC
Module 209	03-Oct-2024	224	164	193	367	CPH, a-IPC, CIC
Module 210	07-Nov-2024	179	92	277	369	CPH, a-IPC, CIC
Total		2,635	1,088	4,487	5,376	

The IPC 200 Series drew a diverse cohort of 2,596 participants, featuring a range of demographic and occupational characteristics, including most registrants were 30–39 (29.8%) years of age, female (77.4%), white (53.7%), Not Hispanic or Latino (63.1%) with 0–2 years in current position (55.2%) and 0–2 years of infection prevention (40.9%) (Table 3). The age distribution of participants was skewed towards younger professionals, with those aged 30–39 years constituting the largest group at 29.8%, followed by 20–29 years at 20.3%, and 40–49 years at 21.6%. The infection prevention and control experience revealed that 55.2% of attendees had been in their current position for 0–2 years, indicating a significant influx of newer professionals into the field. Moreover, 40.9% had 0–2 years of experience in infection prevention, highlighting the introductory level of many participants within the broader context of infection control practice. The gender profile was predominantly female, representing 77.4% of attendees, compared to 20.8% of males and 1.8% of registrants who preferred not to disclose their gender. Racial demographics showed a majority of 53.7% white participants, with Asian and Black or African American attendees comprising 17.3 and 15.1%, respectively. Hispanic or Latino participants accounted for 26.2% of the cohort, while 63.1% identified as not Hispanic or Latino.

**Table 3:** Infection control series attendee demographic and occupational characteristics.

Characteristics	Series (*n* = 2,596)
Age	
<20	7 (0.3%)
20–29	528 (20.3%)
30–29	774 (29.8%)
40–49	562 (21.6%)
50–59	437 (16.8%)
60+	211 (8.1%)
Prefer Not to Answer	77 (3.0%)
Gender	
Female	2,009 (77.4%)
Male	539 (20.8%)
Prefer Not to Answer	48 (1.8%)
Race	
American Indian or Alaskan Native	18 (0.7%)
Asian	448 (17.3%)
Black or African American	393 (15.1%)
Native Hawaiian or Pacific Islander	11 (0.4%)
Two or More Races	81 (3.1%)
White	1,395 (53.7%)
Prefer Not to Answer	250 (9.6%)
Ethnicity	
Hispanic or Latino	679 (26.2%)
Not Hispanic or Latino	1,639 (63.1%)
Prefer Not to Answer	278 (10.7%)
Years in Current Position	
0–2 yrs.	1,434 (55.2%)
3–5 yrs.	689 (26.5%)
6–10 yrs.	243 (9.4%)
10 + yrs.	230 (8.9%)
Years in Infection Prevention	
0–2 yrs.	1,062 (40.9%)
3–5 yrs.	658 (25.4%)
6–10 yrs.	409 (15.8%)
10 + yrs.	467 (18.0%)

Table 4 describes the evaluation of the series’ educational material and its knowledge assessment. Participants in the infection prevention seminar (*n* = 271) rated educational materials as beneficial, with 83.0% strongly agreeing and 16.24% agreeing. Similarly, positive ratings were received for the content presented and its appropriateness for the IPC field, with 85.6% strongly agreeing and 13.3% agreeing that the amount covered was appropriate for the field of infection prevention and control. The clarity and ease of understanding of the material were also highly rated, with 86.7% of participants strongly agreeing that the material was easy to understand and 85.6% strongly agreeing that it was clear and concise.

**Table 4:** Modular post-survey evaluations.

Evaluation questions	*n* (%) (*n* = 271)
The material presented was beneficial?	
Strongly Agree	225 (83.0%)
Agree	44 (16.2%)
Neutral	2 (0.7%)
Disagree	0
Strongly Disagree	0
The amount of content covered was appropriate?	
Strongly Agree	232 (85.6%)
Agree	36 (13.3%)
Neutral	3 (1.1%)
Disagree	0
Strongly Disagree	0
Was the material being easy to understand?	
Strongly Agree	235 (86.7%)
Agree	31 (11.7%)
Neutral	3 (1.1%)
Disagree	2 (0.7%)
Strongly Disagree	0
Was the material clear and concise?	
Strongly Agree	232 (85.6%)
Agree	33 (12.2%)
Neutral	5 (1.9%)
Disagree	1 (0.4%)
Strongly Disagree	0
Was the length of the module appropriate?	
Yes	268 (98.9%)
No	2 (0.7%)
Prefer Not to Answer	1 (0.4%)
Has this material been presented to you before??	
Yes	73 (26.9%)
No	195 (72%)
Prefer Not to Answer	3 (1.1%)
Did the knowledge check activity help improve your understanding of the material?	
Yes	255 (94.8%)
No	14 (5.2%)
Will you implement the knowledge gained from this seminar at your organization?	
Yes	245 (90.4%)
No	5 (1.9%)
Prefer Not to Answer	21 (7.8%)
Do you plan to attend future Infection Prevention Seminar Series modules?	
Yes	268 (98.9%)
No	1 (0.4%)
Prefer Not to Answer	2 (0.7%)

Feedback on webcast logistics showed that 98.9% of respondents found the module length appropriate. Similarly, 72% stated they had not been presented with the material before when asked about familiarity with the material. The practical applications of the seminar were also positively reviewed. A majority (94.8%) confirmed that the knowledge check activity improved their understanding of the material. Furthermore, 90.4% of participants intended to implement the knowledge gained in their organizations, and an equal percentage (98.9%) expressed a desire to attend future seminar modules.

**Figure 1:**
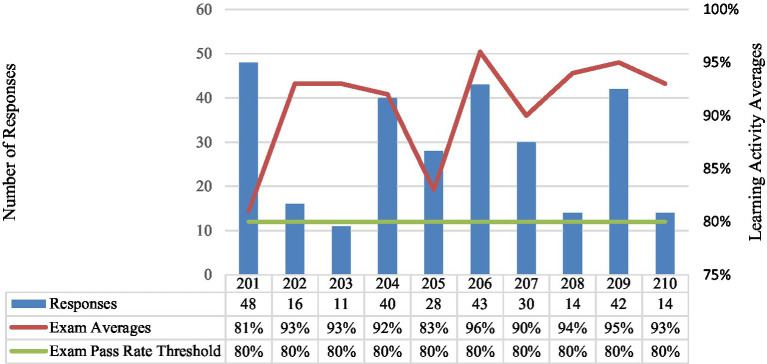
Module learning activity series overview.

The Module Learning Activity Overview (Figure 1) reflects variability in participation and performance across the modules throughout the 200 series. The total number of responses from each module ranged from 11 to 48 individuals completing the activity, with Modules 201 and 204 having the highest participation, at 48 and 40 responses, respectively. Pass rates remain consistently high, ranging from 83% (Module 205) to 96% (Module 206), with most modules achieving pass rates above 90%; recall the threshold pass rate is 80%. While there appears to be a general trend of higher participation correlating with higher pass rates, deviations are observed, such as in Module 205, which exhibits a lower pass rate despite moderate participation.

Participants suggested topics for future sessions. These included outbreak response, surveillance, emerging infectious diseases, and strategies for implementing infection prevention programs in resource-limited settings. These suggestions requested practical, scenario-based learning tailored to real-world applications.

After the series concluded, registered individuals were asked to complete a Level 3 impact survey in December 2024 to measure the series’ impact on infection preventionists. Feedback from the impact survey and the open-ended questions in each module post-survey evaluation provided content needs assessment for the IPC 300 Series. Of the registrants, nine individuals completed the series impact survey. Knowledge checks embedded within the seminars were identified as a valuable learning tool, with 88.9% of participants stating they enhanced understanding. Individuals reported implementing knowledge gained from the series into their practice, with successes including streamlined workflows, improved infection control processes, and reductions in healthcare-associated infections. However, barriers such as resistance to change (25.9%), time constraints (22.2%), and resource limitations (14.8%) were frequently cited, highlighting areas for targeted intervention. Participants emphasized the need for continued development of IPC-related training, particularly in areas such as hospital-acquired infections, enhanced barrier precautions, and data analysis for outbreak investigations, and 88.9% of participants expressed interest in attending future modules, reflecting its role in strengthening infection prevention capacity across diverse healthcare settings.

### Series comparison analysis

A core objective of the program is to conduct ongoing evaluations to ensure it meets IPC community needs and addresses IPC challenges in healthcare settings. One approach to program evaluation was to conduct a comparative analysis of the first-year (100 Series) and second-year (200 Series) modules (11). Both series exhibited varying levels of engagement, with high registration numbers not always translating to high attendance. This trend was consistent across both the 100 and 200 series.

Compared to the 100 Series, the 200 Series demonstrated increased engagement, with YouTube views approximately doubling—from 1,713 views across five modules in the 100 Series to 3,974 views across 10 modules in the 200 Series, a 6.5 times increase as of March 2025. Similarly, registration numbers grew substantially, increasing from a maximum of 284 for Module 104: *Infection Prevention and Control Programs* in the 100 Series to 592 for Module 207: *Outbreaks* in the 200 Series. The per-module YouTube views also increased, with the highest view count rising from 1,713 for Module 105: *National Healthcare Safety Network* to 1,021 for Module 207: *Outbreaks*. Both series’ population demographics were similar, with most registrants being female, constituting over 77% of attendees, with a continuous predominance of White participants. However, the 200 series noted a slight increase in Hispanic or Latino participation from an average of 24.6 to 26.2%, which was insignificant. Post-evaluation responses significantly increased from years 1 to 2 (Table 5) on content appropriateness, understandability, clarity, and conciseness. Years 1 and 2 produced similar responses in implementing material at the organization level and the likelihood to attend future TEPHI IPC modules.

**Table 5:** Comparison analysis between years 1 and 2.

The material presented was beneficial?	
Year 1 Mean	4.77
Year 2 Mean	4.83
*t*-statistic	2.83
*p*-value	0.005
The amount of content covered was appropriate?	
Year 1 Mean	4.69
Year 2 Mean	4.86
*t*-statistic	5.49
*p*-value	<0.001
Was the material being easy to understand?	
Year 1 Mean	4.71
Year 2 Mean	4.87
*t*-statistic	4.93
*p*-value	<0.001
Was the material clear and concise?	
Year 1 Mean	4.84
Year 2 Mean	4.91
*t*-statistic	2.65
*p*-value	0.006
Will you implement the knowledge gained from this seminar at your organization?	
Year 1 Mean	0.898
Year 2 Mean	0.906
*t*-statistic	−0.454
*p*-value	0.65
Do you plan to attend future Infection Prevention Seminar Series modules?	
Year 1 Mean	0.989
Year 2 Mean	0.989
*t*-statistic	0
*p*-value	>1.00

## Discussion

The pilot program offers no-cost infection prevention training tailored for rural and low-resource healthcare facilities, supporting infection prevention professionals in fostering a culture of safety for both healthcare workers and patients (4, 7). Including YouTube as an additional platform significantly expanded the program’s outreach, particularly for Module 207: *Outbreaks*, underscoring the value of online platforms in extending educational content to broader audiences. The 200 Series produced double the content (10 modules compared to 5 in the 100 Series), which coincided with a general trend of higher participant engagement. While the 100 Series provided in-person or virtual attendance options, most participants opted for virtual participation, reporting that it made attendance more flexible and accessible. Consequently, the 200 Series was offered exclusively in a virtual format to better accommodate participant preferences. A comparative analysis of the demographic and occupational characteristics between series reveals consistent trends and subtle shifts within the registrant’s demographics.

Both series demonstrated a significant representation of younger professionals, particularly those aged between 30 and 39 years, indicating a trend toward younger demographics entering the field. Experience levels in infection prevention were predominantly in the 0–2-year range for both series. This underscores a field characterized by high turnover or entry-level engagement seeking additional infection prevention and control training material and resources. Gilmartin et al. discuss similar challenges in the infection prevention workforce, anticipating 40% of IPs retiring in the next 5 years along with individuals suffering from burnout, poor work environments, and a lack of professional opportunities can be influenced (19). This highlights the current and future barriers facilities and infection prevention and control programs will place by having a limited pool of experienced professionals needing to fill a growing gap due to multiple professionals leaving the field. The paper highlighted a variation in comprehensive training and onboarding programs that were not universally implemented or standardized, potentially impacting the preparedness of new hires. Bartles R et al. piloted a beta IP staffing calculator. They determined that most hospitals that participated in the staffing calculator project found that 90% (89.6%, *n* = 277) of hospitals with more than 100 beds were considered to have below expected staffing (20). This, along with staffing shortages, non-consensus of staffing ratios, and the anticipated staffing shortages of IPs, support the creation of easy access to standard training and education programs to help fill this gap.

Infection prevention education and training lack standardization. However, organizations such as APIC, researchers, and practitioners must continuously work to improve the field by developing resources and exploring trends. As a first step, APIC has created multiple professional tools, such as the APIC Textbook and training competencies, to address these challenges within the field (21, 22). More recently, in January 2025, APIC published the Infection Preventionist Academic Pathway (IPAP), a structured educational curriculum designed to support individuals and universities in preparing students for careers in infection prevention and control (23). This initiative aims to collaborate with colleges and universities to develop certificate programs, as well as bachelor’s and master’s degree curricula, tailored to equip individuals pursuing careers in infection prevention and control (23). The IPAP accommodates students at various stages of post-secondary education and includes components such as an Accelerated Internship Program Guide, a Certificate in Infection Prevention & Control, and specialized undergraduate and graduate degree programs (22).

Additionally, an analysis of CIC examination performance over the past decade, including a breakdown of the exam’s eight core competencies, identified specific areas where individuals consistently underperformed. This study indicated that more professionals are becoming credentialed in infection control and epidemiology based on healthcare systems recommendations, job position descriptions requiring certification obtained within a specific timeline, and other regulatory agencies’ environments promoting certification of infection preventionists. This study highlighted the need for more education and training in these areas (24). The consistently lower reliability coefficient values observed in the following topics indicate challenges in assessing these areas: employee/occupational health competencies, management and communication, and education and research (24). Moreover, knowing the low-performing areas of the exam, individual healthcare facilities, systems, and organizations should create robust education based on these knowledge gaps. The multiple organization position paper from SHEA/APIC/IDSA/PIDS highlights the concerns and actions to improve infection prevention and control practices and programs (25). There is an agreement within the field that IPC needs to be a foundational part of an organization’s structure, provided with more resources to be adequately and appropriately resourced, led, and supported (25).

Overall, the seminar series effectively delivered high-quality educational content, as evidenced by the strong positive feedback and the participants’ commitment to apply the knowledge and continue engaging with future offerings (19). The level of participation in learning activities (embedded within the modules) demonstrates the overall effectiveness of the modules in maintaining high pass rates while also indicating areas where participation and performance may require further investigation. When taken all together, this feedback supported continuing the webinar series into year three and including additional topics while simultaneously allowing learners to access course materials from previous modules.

### Future program progression

The series will be extended into a third year. This decision is based on high levels of practitioner participation during webcasts, positive feedback from the second year of the series, and supportive results from the year 1-year 2 module comparison analysis. Year 3 webinars will include a standardized registration process and post-module surveys (implemented at the beginning of year 2), synchronous online delivery (with no in-person option), and an asynchronous option (achieved by recording and posting on YouTube) for learners who are seeking a refresher or were unable to attend the live module in its entirety.

To further enhance registrant numbers and participant engagement, the module format will continue to follow the Year 2 model, featuring sessions of approximately 35–45 min, depending on the topic, followed by a multiple-choice learning activity that incorporates real-life applications, professional experiences, lessons learned, and evidence-based practices. The goal achieved by utilizing learning activities is to foster deeper understanding, encourage discussion among attendees, and provide a metric to assess knowledge gained during the session. Furthermore, it aligns with the requirements of the credentialing organizations that allow TEPHI to offer participants continuing education hours.

The presentation platform will transition to Zoom, from the previously used WebEx and Microsoft Teams platforms, to increase engagement by enabling participants to interact more effectively with the module and fellow attendees during the learning activity. As in Years 1 and 2, feedback regarding the interactive learning activity will be collected through post-event surveys, allowing TEPHI to evaluate activity effectiveness and track trends in improvement and success over time. These adjustments aim to meet the audience’s educational needs and expectations while ensuring continuous refinement of the program’s methodology. To increase audience attendance, the future series will try to obtain additional education hours, such as continuing education hours for physicians (CME) and nurses (CNE). The material could be used in conjunction with physician fellowships that provide educational material on healthcare epidemiology and specifically infection prevention and control program development and oversight.

Obtaining participant feedback has been paramount to the success of this program. Similar input gathered from module-specific post-evaluations and the series impact survey will continue to guide future content development. Moreover, feedback from these sources has consistently highlighted key topics of interest, directly shaping the module topics for year three. Building on the success of the IPC 200 Series, 10 modules will be developed between February and November 2025, each addressing topics identified through participant suggestions. A year-long schedule has been established to maximize the number of registrants and attendance, outlining each session’s topics and corresponding dates.

1. Module 301: Central Line-Association Infections (CLABIs)
2. Module 302: *Clostridioides difficile* Infections (CDIs)
3. Module 303: Infection Prevention and Control (IPC) Training
4. Module 304: Healthcare Environment
5. Module 305: Survey Readiness
6. Module 306: High-Level Disinfection
7. Module 307: Sterilization
8. Module 308: Emerging Infectious Pathogens
9. Module 309: Outbreak Investigation
10. Module 310: Infection Prevention and Control (IPC) in Special Populations

### Limitations and strengths

This program evaluation had several limitations. First, participants’ geographic and demographic representation was somewhat limited. Most participants were female (77%), white (53.7%), and located in Texas (81.4%). This lack of diversity may not fully reflect the broader population of infection prevention or the needs of underserved Texas communities. The transition between delivery platforms, from WebEx® to Microsoft Teams®, may have also introduced participant engagement and experience inconsistencies. The platform change may have caused confusion for participants regarding which platform to use. It’s possible that some previously registered individuals might not have been notified of the switch, requiring re-registration to access the modules. Furthermore, some registrant data was lost during the transition, which may have affected registrant data.

Another limitation was the reliance on self-reported survey data, which introduces potential response bias, as participants may have overstated or understated positive outcomes. Additionally, this analysis focused on short-term outcomes, such as immediate knowledge assessment scores and post-survey ratings, without long-term follow-up to evaluate sustained knowledge retention or practice changes. Kirkpatrick’s Levels 1 (reaction) and 2 (learning) are achieved, while Kirkpatrick’s Level 3 (behavior change) is approached via the impact survey (disseminated at the end of year 2) (10). However, the response rate is too small (*n* = 9) to draw meaningful generalizable conclusions. To address this limitation, the Year 3 pilot program will distribute the impact survey at the six-month mark of the module series and upon completion. This adjusted timeline aims to capture a more comprehensive understanding of long-term knowledge retention and program impact.

Additionally, this program evaluation is subject to several potential biases. Selection bias may have occurred, as participants who registered and completed the surveys will likely be more motivated or engaged, skewing the results toward a more positive evaluation. Response bias is also a concern because participants may have rated the program positively to align with perceived expectations or social desirability. Additionally, while this analysis effectively compared Year 1 (100 series) and Year 2 (200 series) outcomes, the generalizability of the results is limited. Unfortunately, external comparison was not possible because similar programs either do not exist or are not publicly visible. The result, therefore, is that TEPHI was unable to contextualize the program’s outcomes within the broader landscape of infection prevention education.

This analysis demonstrated strengths. For example, TEPHI engaged a large audience and delivered impactful infection prevention education. Participant engagement was evidenced by 1,088 live attendees and over >4,400 YouTube views, underscoring the program’s success in reaching large, diverse audiences, including those in rural and resource-limited settings. Free, widely accessible platforms like YouTube further enhanced the accessibility and participation of individuals throughout the state.

Another key strength of this analysis was its data-driven approach to program design. Comprehensive data collection from registration, attendance, knowledge assessments, and post-module surveys enabled a robust evaluation of program outcomes and informed the development of future modules. A comparative analysis between Year 1 and Year 2 highlighted improved engagement and outcomes, including higher ratings on clarity, content appropriateness, and knowledge application. Additionally, the program aligned with professional certification standards, such as CIC and a-IPC, enhancing its relevance and utility for infection preventionists.

The practical focus of the training modules, incorporating real-life applications, case studies, and scenario-based learning, provided participants with actionable knowledge applicable to their professional settings. Feedback integration further strengthened the program, as participant suggestions directly influenced the development of Year 3 modules. Demographic insights, such as the increasing participation of novice professionals, highlighted the program’s potential to address foundational training needs for those with limited experience in the field.

## Conclusion

These findings demonstrate the growing impact of TEPHI’s Infection Prevention and Control (IPC) training series in addressing critical education gaps for infection preventionists. Year 2 showed increased attendance, YouTube views, and positive feedback, highlighting improved engagement. The program’s alignment with certification standards, practical focus, and responsiveness to feedback enhance its relevance and value.

Areas for improvement remain, including expanding demographic diversity and strengthening competencies in occupational health, communication, and research. Continued adaptation to the needs of novice and experienced professionals, combined with increased accessibility and data-driven improvements, will support the program’s sustained success and broader impact on IPC workforce development.

## Data Availability

The original contributions presented in the study are included in the article/supplementary material, further inquiries can be directed to the corresponding author.
